# Comparative Evaluation of Beverage-Induced Surface Alterations on Dental Enamel: An In Vitro Biomaterial Study

**DOI:** 10.3390/bioengineering13030369

**Published:** 2026-03-22

**Authors:** Ioana Elena Lile, Otilia Stana, Diana Marian, Carolina Cojocariu, Luminiţa Ligia Vaida, Anda Olivia Jesamine Samoilă, Iustin Olariu

**Affiliations:** 1 Department of Dentistry, Faculty of Dentistry, “Vasile Goldis” Western University of Arad, 94-96 Revolutiei Blvd., 310025 Arad, Romania; lile.ioana@uvvg.ro (I.E.L.); stana.otilia@uvvg.ro (O.S.); olariu.iustin@uvvg.ro (I.O.); 2 Department of Dentistry, Faculty of Medicine and Pharmacy, University of Oradea, 1 Universitatii Street, 410087 Oradea, Romania; 3 Multidisciplinary Doctoral School, Vasile Goldis Western University of Arad, 310414 Arad, Romania; samoila.anda@uvvg.ro

**Keywords:** dental erosion, enamel demineralization, beverage acidity, staining, dietary acids, chlorhexidine, oral health

## Abstract

Background/Objectives: Despite advances in preventive dental care, tooth enamel erosion remains a relevant concern, and very few comparisons of surface topography have been carried out under controlled conditions in the laboratory. This study primarily aimed to conduct a qualitative morphological evaluation, supported by semi-quantitative image analysis, of the effects of commonly consumed beverages on human enamel morphology and colour, and to explore their relationship with beverage acidity in an in vitro model. Methods: Forty-two human teeth were allocated at random into seven different groups, each containing six molars. These groups were Coca-Cola, orange juice, lemon juice, coffee, chlorhexidine, regular mouthwash without chlorhexidine, and one control group. Following a 24 h exposure to a simulated saliva environment at 37 °C, the test samples were then subjected to a five-day erosion cycle. SEM analysis was used to examine the enamel alterations after evaluating the morphology of the enamel surface and by digital image analysis. Results: Scanning electron microscopy, SEM, showed how erosion of the teeth’s surface increased with the acidity of the drink. The extensive exposure of the crystal prisms, along with the severe loss of intercrystalline material and honeycomb weathering patterns, was all brought about by Coca-Cola and lemon juice. The moderate erosion brought on by orange juice in tests resulted in partially exposed prisms. Both the mouthwashes and the coffee exhibited similar impacts on the tooth enamel in a microscopic view. Minimal enamel prism rods were exposed due to either the coffee or the mouthwash. The surface characteristics were found through a digital image analysis, which indicated alterations in surface texture. Conclusions: Under these immersion conditions, highly acidic beverages produced the most pronounced enamel surface changes, whereas coffee induced mainly staining and neutral mouthwashes caused minimal modification. These results reflect qualitative morphological trends and should not be interpreted as clinical outcomes.

## 1. Introduction

Dental enamel is essential for protecting teeth from chemical, mechanical, and microbial damage. Once enamel is formed, it cannot regenerate and can thus suffer progressive wear over the years and permanent weakening. Its structural stability may be affected by various intrinsic and extrinsic factors, including dietary behavior, age-related changes, and clinical interventions. It has been demonstrated in previous studies that the mineral density and crystallinity of enamel can be affected by environmental factors [1], that the reduction in enamel in the interproximal area can result in surface irregularities of varying depth [2], and that bioactive-glass sealants can help inhibit demineralization [3]. Taken together, these studies underline enamel’s multifactorial vulnerability to biological and environmental insults.

Dietary behavior is one of the main determinants of enamel health. Acidic foods and drinks have the greatest effect on chemical erosion; sugary and sour products amplify this process by favoring demineralization. Remineralising strategies, including casein phosphopeptide–amorphous calcium phosphate (CPP–ACP) complexes [4] and fluoride-based agents [5], have been shown to support enamel stabilisation following acid exposure; however, such clinical preventive measures are outside the scope of the present in vitro model. Although beverage pH is commonly used as an indicator of erosive potential, enamel dissolution is influenced by multiple physicochemical factors, including titratable acidity, buffering capacity, chelating properties of organic acids, and mineral ion content. These beverages were selected because they represent commonly consumed acidic drinks and two widely used mouthwash formulations, allowing comparison across products of high clinical and epidemiological relevance [6,7,8,9,10,11].

Experimental and clinical evidence demonstrate that both soft drinks and fruit juices increase enamel surface roughness and promote demineralization, with carbonated beverages exhibiting the most significant erosive potential [12,13,14]. Dental erosion, defined as the chemical loss of dental hard tissues without bacterial involvement, is particularly prevalent among young individuals due to frequent consumption of acidic drinks [15]. pH and titratable acidity are now recognized as the main predictors of erosive risk [8,10]. Moreover, toothbrushing immediately after acid exposure can exacerbate enamel loss, highlighting the multifactorial nature of erosion [8,16]. Since this process is irreversible, preventive measures such as topical fluoride and remineralising formulations are vital.

The pH level for most carbonated beverages is below the critical demineralization level of 5.5 [17]. Under scanning electron microscopy studies, acidic drinks—citrus juices included—have been shown to cause surface cracking, roughening, and substantial enamel loss [18,19,20,21]. Although fruit juices or herbal beverages are widely perceived as “natural” and safer substitutes, several studies show that they can lead to enamel loss equal to or greater than that produced by carbonated sodas [19]. The erosive effect is primarily attributed to the combined impact of low pH, prolonged exposure, and insufficient buffering or calcium content [20,21]. While prevention measures have promoted increased public awareness, enamel lesions persist, indicating that behavioral and dietary factors still make an impact.

Systematic analysis and detailed description of the ultrastructure of enamel using scanning electron microscopy have been lacking; typically, the microscopy has been utilised descriptively, without quantitative analysis.

Study Rationale and Objectives was to overcome these methodological shortcomings by: implementing a pH cycling protocol which includes periodic acid challenges and periods for remineralization through artificial saliva to simulate more real-world oral conditions better, using standardised SEM imaging with systematic morphological evaluation, and digital image analysis for a semi quantitative assessment of the degree of erosion and to make a comparison of commonly consumed beverages in a controlled environment to assess the relative risk ranking of these drinks. The objective was to evaluate and compare the effects of erosion and staining on human teeth caused by the commonly consumed drinks: cola, orange juice, lemon juice, coffee, chlorhexidine mouthwash, and a chlorhexidine-free mouthwash, using a model that alters the pH and SEM assessment. The patterns of tooth surface loss produced by various drinks need to be determined, so our initial investigation aimed to correlate erosion severity with beverage pH and acid type.

Understanding the erosive potential of commonly consumed beverages remains clinically relevant. Controlled in vitro models allow reproducible evaluation of enamel surface alterations under standardized exposure conditions and provide mechanistic insights that may support preventive dietary recommendations in clinical practice. Previous in vitro studies have demonstrated that controlled acidic exposure models provide reproducible insights into enamel erosion mechanisms and surface alterations [22,23,24].

Based on these considerations, the present study aims to qualitatively compare enamel surface morphology and staining after exposure to six commonly consumed beverages, addressing the need for standardised descriptive data under a controlled in vitro pH-cycling model.

## 2. Materials and Methods

The present in vitro study aimed to evaluate the erosive and staining effects of six commonly consumed beverages on human dental enamel. A standardized methodology was applied throughout to ensure reproducibility, reliability, and control of experimental variables.

### 2.1. Sample Collection and Preparation

A total of 42 extracted third molars were included in the study. The specimens were randomly assigned to seven groups (n = 6 each), consisting of six experimental groups exposed to the test beverages and one control group maintained in artificial saliva (modified Fusayama–Meyer artificial saliva buffered with HEPES). The tooth specimens were third molars, extracted by dentists for orthodontic purposes in the previous 2 weeks and collected from donors aged 18 to 25 years for the study. All teeth exhibiting fissures, carious lesions, or structural damage were removed by the research team. Specimens needed cleaning to remove soft tissue fragments and were then transferred into 0.1% thymol solution at 4 °C to prevent bacterial growth and drying. The specimens were kept in leak-proof containers at 4 °C until use, and all procedures were performed within 2 weeks of tooth extraction to ensure preservation of enamel structure. Although 0.1% thymol is widely used as an antimicrobial storage solution in dental research, such solutions can influence enamel properties. Studies indicate that short-term storage (≤2 months) in 0.1% thymol maintains acceptable enamel microhardness [25], whereas extended storage may alter enamel structure [26,27]. In this study, all specimens were stored for no longer than 2 weeks and processed using identical protocols, ensuring consistent storage effects across all groups.

Specimens from different donors were randomly distributed across the seven groups, including the control group, to reduce donor-related variability in enamel morphology and composition. Nevertheless, biological heterogeneity could not be eliminated and is acknowledged as a limitation of the study.

### 2.2. Experimental Groups and Test Solutions

The specimens were randomly assigned to six experimental groups and one control group (n = 6 per group). The experimental groups corresponded to the following test solutions (Table 1): Coca-Cola, lemon juice, orange juice, coffee, mouthwash with chlorhexidine, and mouthwash without chlorhexidine. The control group was immersed exclusively in artificial saliva (modified Fusayama–Meyer artificial saliva buffered with HEPES) under identical temperature and cycling conditions.

Before use, all test solutions had their pH levels determined by a pH meter, which had been calibrated at ambient temperature (23 ± 2 °C). Every day, the pH meter (HI98107 pHep, Hanna Instruments, Cluj-Napoca, Romania) was calibrated using standard buffer solutions with pH levels of 4.0, 7.0, and 10.0. The different available enamel surface areas required 6 specimens per group (n = 6). The six tested substances were commercial solutions. The experiment employed one control solution, artificial saliva (modified Fusayama–Meyer artificial saliva buffered with HEPES; pH 6.8–7.0).

The specimens underwent pH cycling, which simulates conditions in the mouth. The specimens followed a pH-cycling protocol to imitate the occasional acidic challenges found in the mouth. For each erosion cycle, they were placed in separate 20 mL polypropylene containers with the test beverage, making sure the enamel surface faced up. Each specimen was fully submerged in the test solution during the immersion cycles to ensure uniform exposure of the enamel surface. Each immersion lasted 90 s at 37 ± 1 °C in a water bath with gentle shaking at 60 oscillations per minute to mimic how saliva moves and to avoid stagnant layers. After each cycle, the specimens were taken out, rinsed with 10 mL of deionised water for 10 s to remove any leftover beverage, and gently dried with absorbent paper before starting the next cycle.

Since each specimen was placed in a much larger volume of solution (20 mL) than the enamel surface area, major changes in the overall pH during the short exposure were unlikely. Still, real-time pH monitoring was not done, so small local pH changes at the enamel–solution interface may have occurred.

However, the specimens were first subjected to a pre-treatment stage in which they were immersed in an artificial saliva solution. This solution consisted of 1.5 millimolar calcium chloride, 0.9 millimolar potassium dihydrogen phosphate, 130 millimolar potassium chloride, and 20 millimolar HEPES, with a pH of 7, at 37 °C for 24 h. The erosion protocol comprised 4 challenges with acidic drinks per day, lasting 90 s at 23 ± 2 °C. This was followed by 2 h of remineralisation in fresh saliva at 37 °C. Samples were left overnight in fresh saliva at 37 °C after daily testing on a cyclical regimen. Twenty erosive challenges were accomplished over five days through the same process. This simulation more closely replicates the human oral environment by incorporating periods of remineralisation during which saliva can interact with the tooth surface between drinks.

### 2.3. Experimental Design and Immersion Protocol

Each enamel sample was provided with its own 20 mL container, containing one of six test solutions. Solutions were labeled according to their contents: lemon juice (RL), Coca-Cola (RCC), orange juice (RP), coffee (RC), chlorhexidine mouthwash (RAC), and chlorhexidine-free mouthwash (RA). Coca-Cola (The Coca-Cola Company, Atlanta, GA, USA) was used fresh from bottles that were newly opened and kept at 4 °C. While some studies remove carbonation from beverages to avoid bubble adhesion [28], this study used carbonated Coca-Cola to better reflect real-world conditions, since carbonation is present when people typically drink it. During immersion, specimens were gently agitated to make sure the acid contacted the enamel evenly and to stop bubbles from collecting on the surfaces. The containers were sealed and maintained at 37 °C to approximate intraoral temperature. To maintain chemical stability and prevent contamination, every solution was replaced daily. To simulate the drinking and waiting process, a pH cycling model was used. On day zero, the initial pre-treatment phase involved suspending the test specimens in 20 mL of saliva analog solution at 37 °C for 24 h in individual beakers, which were gently agitated at 60 revolutions per minute. The pre-treatment step allowed minerals such as phosphate and calcium to form a layer on the enamel surface, similar to the acquired dental pellicle that forms in the mouth. For each of the first five days, the daily routine was as follows: the day was divided into four immersion cycles, each taking place at 9 am, 12 pm, 3 pm, and 6 pm. During both acidic challenges and remineralization phases, specimens were subjected to gentle agitation at 60 rpm to ensure uniform solution contact and to minimize boundary layer formation at the enamel surface. The specimens were removed from the artificial saliva. The samples were rinsed in deionised water for five seconds. Placed in 20 mL of fresh test solution at a temperature of 23 ± 2 °C. The duration was 90 s, with agitation extremely gentle at 60 revolutions per minute. They were then gently rinsed for 5 s with deionised water. The specimens were submerged in 20 mL of fresh artificial saliva at 37 °C. The specimens were left for 2 h at 37 °C in an artificial saliva solution. Acidic immersion cycles were performed at 23 ± 2 °C to simulate beverage consumption temperature, whereas remineralization phases in artificial saliva were maintained at 37 °C to approximate intraoral conditions. After each test, the artificial saliva was replaced with a newly prepared sample. After the final challenge, the test specimens were stored overnight in fresh 37 °C artificial saliva until the next morning. The storage took place from 6 pm until 9 am the following day. Overall, this procedure involved five days of cycling. Each day consisted of four immersion cycles. A total of 20 erosive challenges were performed. Each challenge was timed at 90 s. Over five days, cumulative exposure to acidic conditions was thirty minutes. The experiments were carried out in a controlled laboratory environment at 23 ± 2 °C. The saliva was incubated in a 37 °C water bath. To prevent cross-contamination, each sample was stored in a separate container. A detailed record was kept of the timing of each procedure. The test substances used represent a moderate frequency of consumption. Specimens were taken from the artificial saliva. Rinsed with deionised water for 10 s. Dried in air at room temperature for twenty four hours.

### 2.4. Evaluation of Enamel Alterations

#### 2.4.1. Scanning Electron Microscopy

After being dried, samples were secured to aluminium studs by using tabs coated with carbon. Using a sputter coater (Quorum Q150R ES, Quorum Technologies, Laughton, East Sussex, UK ), the samples were gold-palladium coated to a thickness of 5 nm for 60 s under the following conditions: Current: 20 mA and vacuum: 0.1 mbar. This was done within an argon atmosphere. Samples were then observed in the SEM under the following conditions: all observations were made on a FEI Quanta 250 (FEI Company, Hillsboro, OR, USA). The study utilised an Everhart-Thornley detector (which captures secondary electrons) to observe coated tooth specimens. The samples were coated with metal beforehand, resulting in topographic images of the defect using low-voltage electron beams and a small electron probe to prevent sample surface distortion and preserve surface detail, with beam deceleration mode. Higher voltages were used to accelerate the process on samples that produced weak signals. To obtain the images, a digital camera was used to photograph the buccal surfaces of the specimens. All images were centered on the middle third of the buccal enamel surface to ensure consistency. Each sample consisted of photographs taken of the area. Also, pictures were taken of specific erosion features in the samples, and the microscope was meticulously focused at all magnifications used. Astigmatism was corrected in the system, and brightness and contrast were then optimised for each image. The experimenters checked several regions on each sample to ensure that the images taken were representative.

Tooth wear was assessed using the Basic Erosive Wear Examination index developed by Bartlett et al., a groundbreaking system [29]. The BEWE-based grading was used solely as a descriptive framework to facilitate comparison with existing clinical literature, not as a direct surrogate for clinical scoring, on how the wear progressed through six distinct enamel loss stages ranging from enamel that has not been affected (grade 0, BEWE 0) to dentine exposure (grade 5, advanced BEWE 3). Grade 0 was defined as an enamel surface indistinguishable from the unexposed baseline control, with no evidence of surface modification following immersion. Table 2 shows the changes in ultrastructure associated with each BEWE score investigated by scanning electron microscopy on extracted enamel samples, which were then graded according to a six-stage scale of enamel appearance:

Grade 0—no erosion;—corresponding to BEWE 0
Grade 1—minimal erosion; corresponding mainly to BEWE 1
Grade 2—mild erosion; corresponding to BEWE 1–2
Grade 3—moderate erosion; corresponding to BEWE 2
Grade 4—severe erosion; corresponding to BEWE 3
Grade 5—extreme erosion; corresponding to advanced BEWE 3 with dentine exposure

The evaluation procedure consisted of independent assessments of all SEM images by two people (E1 and E2), who had been calibrated beforehand by assessing typical test images. The examiners were unaware of which groups the specimens belonged to. The images were shown to the subject in a random order. The examiners used a grading scale of 0 to 5 to assign erosion levels. Agreements were confirmed after all discrepancies were resolved through a consensus discussion. The agreement between observers was evaluated using Cohen’s kappa statistic. Inter-examiner reliability for SEM grading was assessed using Cohen’s kappa coefficient. The agreement between evaluators was substantial, with a kappa value of κ = 0.82 (95% CI: 0.74–0.90), indicating high grading consistency according to Landis and Koch criteria. Before conducting the formal assessment, the examiners viewed 15 images representing the range of erosion grades. They discussed their classification criteria until they reached an agreement of more than 90% on examples of these images.

#### 2.4.2. Macroscopic Analysis

The researchers performed macroscopic analysis by using standardized visual examination of the tooth specimens under controlled lighting conditions. The researchers documented all observed color changes, surface texture modifications, and erosive defects using photographic methods. All the specimens were examined at magnifications ranging from ×1.0 to ×2.7 using a KAPS 1100 stereomicroscope (KAPS 1100, Karl Kaps GmbH & Co. KG, Asslar, Germany). The researchers determined surface irregularities and fissures, discoloration and porosity, and deposit formation through microscopic analysis.

### 2.5. Data Analysis

Sample size was estimated a priori using G*Power 3.1 software (Heinrich Heine University, Düsseldorf, Germany) for a one-way ANOVA design including seven experimental groups. Based on pilot observations obtained from three specimens per group, which indicated marked morphological differences between highly acidic beverages (Coca-Cola and lemon juice) and near-neutral solutions (control and mouthwash groups), a large effect size was assumed (Cohen’s f = 0.50). With a significance level of α = 0.05 and a desired statistical power of 0.80, the minimum required total sample size was calculated as 35 specimens (five per group). To increase robustness and account for potential specimen loss during preparation or analysis, six specimens per group were included, resulting in a total sample size of 42 specimens.

Descriptive comparisons among the six experimental groups were performed, with the control group maintained in artificial saliva serving as the baseline reference. Continuous variables were summarised as mean ± standard deviation or median (range), while ordinal variables were presented as frequencies and percentages. Inter-examiner agreement for SEM grading was assessed using Cohen’s kappa coefficient. Because the study was designed primarily as a qualitative morphological evaluation supported by semi-quantitative image analysis, inferential statistical comparisons between groups were not performed.

### 2.6. Ethical Considerations

The study protocol was reviewed and approved by the Institutional Ethics Committee of the Faculty of Dentistry, “Vasile Goldiș” Western University of Arad (Approval No. 20/2025). All procedures conformed to the ethical standards of the Declaration of Helsinki for research involving human tissue.

**Figure 1:**
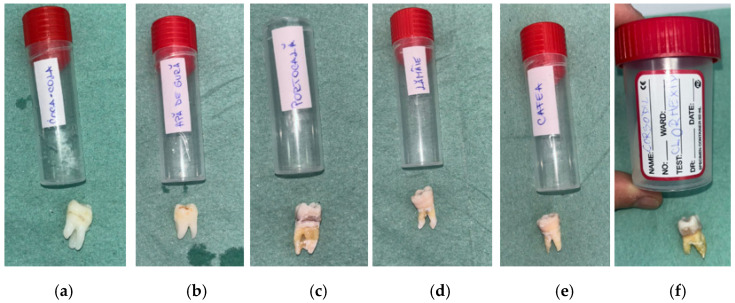
Labeled containers and corresponding tooth specimens assigned to each experimental group before immersion: (**a**) Coca-Cola (RCC); (**b**) mouthwash without chlorhexidine (RAC) (**c**) orange juice (RP); (**d**) lemon juice (RL mouthwash chlorhexidine (RA); (**e**) coffee (RC); (**f**) mouthwash with chlorhexidine (RAC).

Before beginning the immersion process, the researchers placed each enamel sample into a separate test group and positioned it adjacent to the container containing the intended solution. Figure 1 shows the initial appearance of human molars and the containers used for each solution. It also illustrates how the samples were spread out before they were shown.

## 3. Results

The images at the macroscopic and stereomicroscopic levels show changes in enamel after cyclic erosive exposure. These should be taken strictly as a qualitative illustration, not as something that would occur in actual clinical conditions.

Control observations: No observable macroscopic or microscopic alterations occurred in the specimens kept in artificial saliva or distilled water. These samples served as baseline references and were therefore not included in the erosion severity comparison.

### 3.1. Experimental Specimen Standardization

Prior to experimental procedures, extracted human molars were cleaned of soft tissue debris. The enamel surfaces were gently cleaned and visually inspected to exclude specimens presenting cracks, caries, or structural defects. Comparable flat enamel regions were selected for all samples. A standardized exposure window was defined on the buccal enamel surface, while the remaining tooth structure was protected to ensure uniform contact with the test solutions. All specimens were randomly allocated to experimental groups to minimize baseline variability. No mechanical polishing was performed in order to preserve the natural enamel surface morphology.

Each experimental group consisted of six independent tooth specimens (n = 6), and all analyses were performed on individual samples to ensure biological replication. SEM imaging and quantitative ImageJ (Version 1.53t) measurements were conducted on standardized regions of interest for each specimen.

### 3.2. Macroscopic Observations

**Figure 2:**
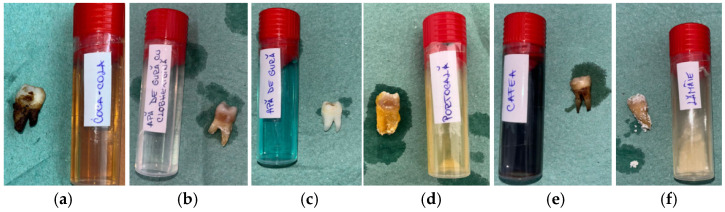
Macroscopic appearance of enamel specimens after 5 days: (**a**) Coca-Cola (RCC)—brown discoloration and marginal staining; (**b**) mouthwash with chlorhexidine (RAC)—thin whitish residue at the cervical area; (**c**) mouthwash without chlorhexidine (RA)—slightly whitened surface (RA); (**d**) orange juice (RP)—chalky, opaque crown surface; (**e**) coffee (RC)—brown surface staining; (**f**) lemon juice (RL)—whitish mineral deposits on crown and root.

The macroscopic evaluation after 5 days showed clearly visible differences between the groups (Figure 2). The samples in Coca-Cola and citrus juices showed the earliest signs of surface dullness and discoloration, which deepened with time. Those treated with Coca-Cola had a yellow–brown surface staining, especially in the cervical regions, whereas exposure to lemon juice resulted in whitish, chalky spots that resembled surface softening. The specimens in orange juice developed opaque areas, and the surface gloss was lost. Coffee caused mostly uniform brown staining without any significant surface breakdown. The chlorhexidine-free mouthwash-exposed samples were almost the same, and the chlorhexidine-containing mouthwash gave rise to thin white deposits around cervical margins.

### 3.3. Microscopic Observations

**Figure 3:**
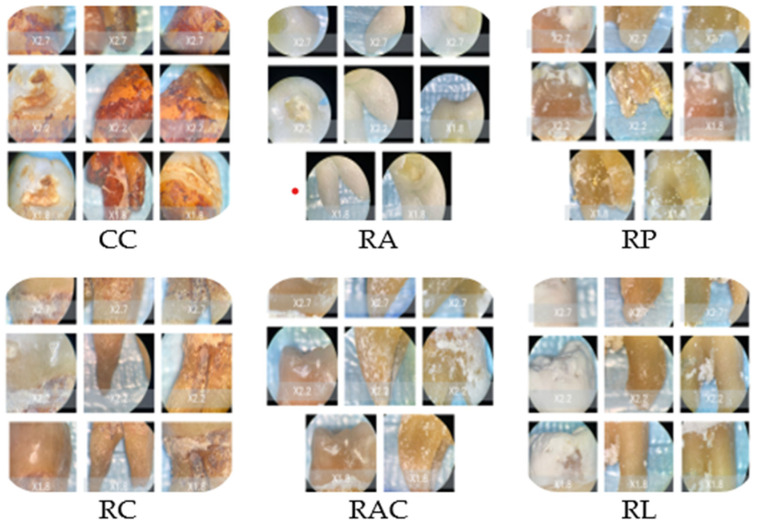
Representative stereomicroscopic images (×1.0–×2.7 magnification) illustrating macroscopic enamel surface changes following immersion in the tested solutions. CC: Coca-Cola group showing evident surface discoloration, loss of surface gloss, and irregular erosive patterns consistent with acid-induced mineral dissolution. RC: Coffee group characterized predominantly by extrinsic staining deposits with limited visible structural surface alteration. RA and RAC: Mouthwash groups showing minimal macroscopic modification, with surfaces remaining comparable to baseline enamel morphology. RP: Orange juice group displaying moderate surface opacity and localized erosive changes indicative of intermediate enamel dissolution. RL: Lemon juice group exhibiting extensive surface opacity, chalky white areas, and heterogeneous texture suggestive of advanced demineralization.

The stereomicroscopic evaluation (Figure 3) revealed marked morphological differences between the groups. Samples from Coca-Cola showed surface cracking, were porous, and were heavily stained to a great depth. The lemon juice treatment caused the surface to become rough, the sample to become porous, and a significant loss of mineral content. The orange juice led to the formation of pits and the roughening of the enamel with a small area of discoloration. Coffee-treated samples revealed only slight surface changes and pigment deposition. The mouthwash with chlorhexidine caused the surfaces to be slightly stained and changed to a minimal extent, while the chlorhexidine-free mouthwash-treated surfaces looked like the baseline ones. Macroscopic evaluation revealed progressive enamel surface alteration ranging from preserved morphology in control samples to pronounced opacity and surface irregularities in highly acidic solutions (Figure 3).

### 3.4. Scanning Electron Microscopy Findings

**Figure 4:**
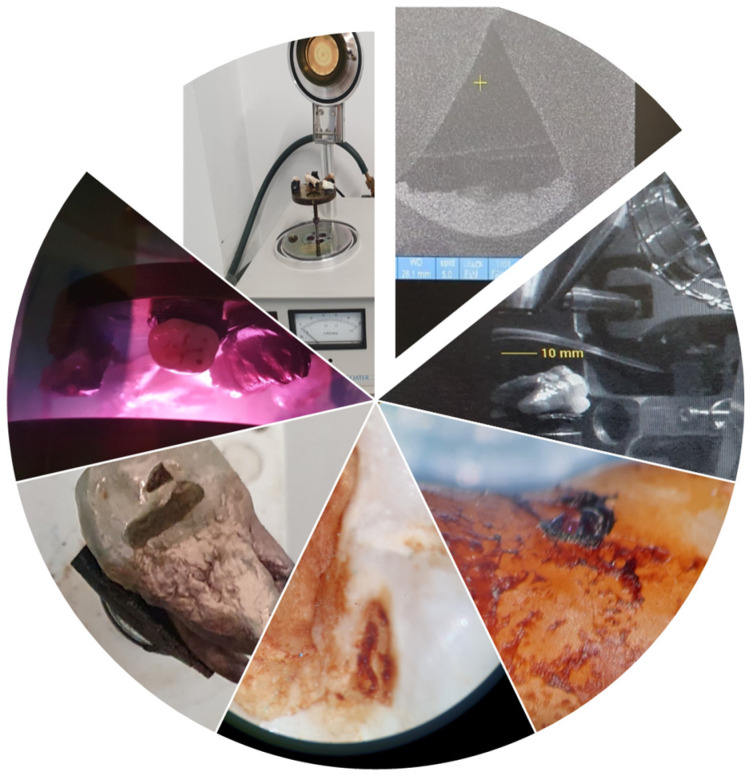
SEM procedure assessment.

SEM analysis demonstrated a correlation between the enamel surface texture and the acidity of the beverage tested (Figure 4). Chlorhexidine-free mouthwash and chlorhexidine mouthwash with a pH ranging from 5.52 to 6.81 had enamel surfaces that appeared relatively smooth with minimal structural changes. Boundaries of the prisms were barely visible even at high magnification, and the interprismatic substance remained largely intact. Coffee with a pH of 5.18 had an enamel structure well-preserved with only minimal prism boundary visibility, associated with superficial surface modification and staining deposits. Surface staining was evident as organic deposits, but underlying enamel architecture remained intact. Orange juice with a pH of 4.78 had moderate enamel surface changes. Prism boundaries became clearly visible, with partial exposure of prism cores and moderate loss of interprismatic substance, creating an early honeycomb pattern.

Coca-Cola, with a pH of 2.52, showed severe erosion patterns. Extensive prism exposure with significant interprismatic substance loss created a pronounced honeycomb appearance. Deep erosive craters were visible, and prism cores showed signs of dissolution. Lemon juice, with a pH of 3.70, produced erosion patterns comparable to those observed for Coca-Cola, including extensive prism destruction and loss of interprismatic substance. The surface showed deep irregular patterns, exposed subsurface enamel layers, and, in some areas, loss of prism architecture. SEM images confirmed that the beverages associated with the most pronounced erosion were Coca-Cola and lemon juice. Scanning electron microscopy revealed that tooth damage increased with the acidity of the drinks.

**Figure 5:**
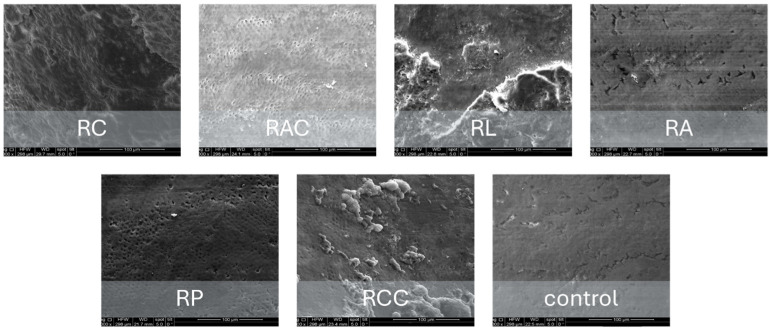
Representative SEM micrographs illustrating enamel surface morphology following exposure to the tested solutions. Surface morphology was evaluated by SEM at ×1000 magnification under an accelerating voltage of 20 kV, using an Everhart–Thornley detector in high-vacuum conditions. Control samples exhibited intact prism structure, while acidic beverages induced progressive interprismatic dissolution and prism exposure, most pronounced in lemon juice and Coca-Cola groups. Mouthwash and coffee groups showed minimal morphological alteration.

Representative SEM micrographs illustrating enamel surface morphology after exposure to the tested solutions are shown in Figure 5. 

### 3.5. Quantitative Image Analysis

Quantitative grayscale analysis of SEM images was performed using ImageJ software to objectively assess enamel surface texture changes. Progressive increases in grayscale variability (StdDev gray value) were observed from the control and mouthwash groups toward acidic beverages. Lemon juice and Coca-Cola demonstrated the highest texture variability, indicating pronounced surface disruption, whereas mouthwash solutions showed values comparable to baseline enamel conditions. These quantitative findings (Table 3) supported the qualitative SEM observations.

**Figure 6:**
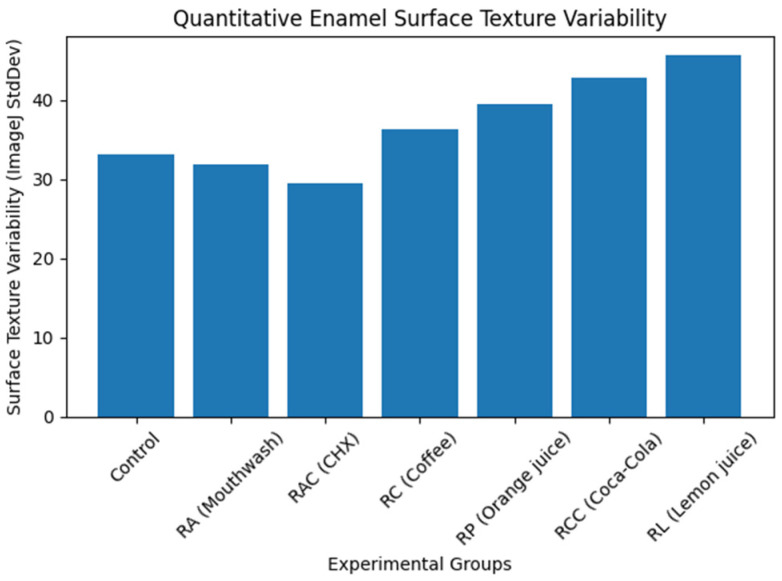
Quantitative enamel surface texture variability derived from SEM image analysis (ImageJ). Surface texture variability (StdDev gray value) increased progressively from the control and mouthwash groups toward acidic beverages, indicating increasing enamel surface irregularity and erosive damage.

Visualization of grayscale texture variability (Figure 6) demonstrated a progressive increase in enamel surface heterogeneity from control and mouthwash groups toward acidic beverages, with lemon juice showing the highest values.

### 3.6. Distribution of Descriptive Grading Categories

A semi-quantitative grading system has been used to assess enamel surface alterations associated with the BEWE score, with levels ranging from grade 0 to grade 5; these grades equate to increasing levels of erosion. No specimens from the experimental solution groups were classed as grade zero. Minimal erosion (grade 1; mainly corresponding to BEWE 1) was recorded exclusively in the chlorhexidine-free mouthwash group. Mild erosion (grade 2; corresponding to BEWE 1–2) occurred only in the chlorhexidine-containing mouthwash group. Moderate descriptive grading (grade 3; corresponding to BEWE 2) was identified in the coffee group, reflecting increased prism boundary visibility and surface texture alterations rather than substantial structural enamel loss. In the coffee group, the assigned grade primarily reflected superficial morphological changes and staining-related surface texture variation detectable under SEM, while the underlying enamel architecture remained largely preserved. Therefore, this grading should be interpreted as descriptive rather than indicative of clinically relevant erosion. Severe erosion (grade 4; corresponding to BEWE 3) was observed in all specimens exposed to orange juice. The most extreme erosion (grade 5; corresponding to advanced BEWE 3 with dentine exposure) was detected in all Coca-Cola and lemon juice samples. Grade 3 corresponded to localized prism exposure and partial surface demineralization, whereas Grade 4 was assigned when prism dissolution and interprismatic loss were generalized across the analyzed surface area, indicating advanced erosive damage. The findings of this experiment show different erosion patterns on the enamel surfaces exposed to various beverages over an extended period.

### 3.7. Descriptive Relationship Between pH and Observed Damage

The allocation of grading categories closely reflected the acidity of the tested beverages (Table 4). Materials with lower pH (Coca-Cola, lemon juice, orange juice) were associated with higher descriptive damage categories. In contrast, drinks with a pH close to neutral (mouthwashes) showed very few changes. This pattern reflects general tendencies of morphological change under the experimental conditions and should not be interpreted as a quantitative measure of erosion.

Quantitative grayscale texture analysis supported the qualitative SEM grading, confirming increasing surface heterogeneity with increasing beverage acidity.

## 4. Discussion

All results of this study should be viewed in light of an ongoing, non-physiological in vitro immersion model, which is known to increase erosive effects compared to sporadic oral exposure. The study aimed to qualitatively compare how six beverages caused tooth erosion and staining. The novelty of this study lies in combining qualitative SEM morphology with reproducible semi-quantitative grayscale analysis within a controlled cyclic erosive exposure model, providing an intermediate methodological framework between purely descriptive microscopy and fully mechanical surface characterization. The present findings should not be interpreted as a direct consequence of pH alone. Citric-acid–containing beverages may exhibit disproportionately high erosive potential due to calcium chelation and elevated titratable acidity, whereas phosphoric-acid beverages may differ in buffering behavior and mineral interactions. Because titratable acidity and mineral composition were not assessed, the current results describe comparative morphological trends rather than complete physicochemical mechanisms.

These findings must be interpreted within the limitations of a cyclic erosive exposure model representing a non-physiological in vitro condition that may produce more pronounced alterations than those occurring intra-orally. Within this experimental framework, acidic lemon juice and Coca-Cola (pH ≤ 3.8) produced the most evident enamel surface alterations, whereas orange juice (pH 4.8) induced noticeable but comparatively less extensive changes.

Coffee consumption resulted only in extrinsic staining, with minimal surface modification. Both mouthwash formulations—especially the neutral chlorhexidine-free product—showed practically no enamel effects. The semi-quantitative grading scale captured subtle ultrastructural surface alterations detectable under SEM that may not correspond to clinically significant enamel erosion. These results are consistent with previously described associations between beverage acidity and enamel demineralisation. However, they should not be interpreted as confirming clinical behaviour because the experimental exposure does not replicate intraoral conditions. Nonetheless, as the exposure inside the mouth is of an intermittent nature and is very much dependent on saliva, pellicle formation, and natural pH changes, the present findings ought to be regarded as a ranking of the different agents in terms of their erosive potential rather than a direct depiction of clinical outcomes. This is consistent with previous findings that pH and titratable acidity are strong determinants of erosive capacity [6,7,8,10,17,21]. The current results should be seen as qualitative ones, which is a point that cannot be overlooked. Indeed, population-based studies have demonstrated that oral hygiene behaviours and the use of adjunctive cleaning methods significantly influence plaque accumulation and gingival health, with notable differences between urban and rural young adult populations in Romania, underscoring the multifactorial determinants of oral surface changes and the inherent limitations of isolated in vitro models [30]. The surface of the enamel was observed in great detail with a scanning electron microscope after being subjected to pH cycling. Extensive structural degradation of these specimens was observed in acidic drinks, while near-neutral pH beverages showed minimal degradation. This progressive damage to the teeth was found to be strongly linked to the drinks’ pH levels. Previous SEM analyses have demonstrated that the honeycomb structure seen in samples treated with Coca-Cola and lemon juice is consistent with the erosion of the enamel interprismatic matrix. SEM was able to differentiate between surface staining and the loss of tooth structure. It thus demonstrated that the effects of chlorhexidine and coffee are distinct from those of the loss of structure.

The steep increase in damage scores as pH dropped below 4 was consistent with earlier research showing significant enamel softening and mineral loss under low pH and high titratable acidity conditions [6,10,17]. The destructive effects of lemon juice and Coca-Cola reflect previous reports that citrus-based beverages and colas represent some of the most erosive dietary acids [6,7,9,12,13,17,18,19,20,21]. Citric acid, the main acid component of lemon and orange juices, binds calcium and chelates extensively and is a factor in prolonged demineralisation as it inhibits remineralisation [6,7,21]. In addition to calcium chelation by citric acid, enamel dissolution is also influenced by the saturation state of calcium and phosphate ions in the surrounding solution. Beverages that are undersaturated with respect to hydroxyapatite promote mineral loss, whereas solutions approaching calcium–phosphate saturation may partially reduce the erosive potential. Because the mineral ion composition of the tested beverages was not determined in the present study, the contribution of saturation effects could not be evaluated directly. Both beverages presented high-grade damage, fissuring, porosity, and deep pigment penetration (9/10) in the current study, emphasising the significant erosive potential with repeated exposure. Orange juice also displayed significant erosive effects (8/10), generating large surface roughening, pitting, and chalky mineral loss, confirming previous reports in the literature that fruit juices are at least as erosive as or more so than carbonated sodas [20,21]. These effects are probably due to their pH, organic acid, and intrinsic calcium contents [7,20,21].

Exterior discolouration caused primarily by coffee (pH ≈ 5.0) is consistent with previous reports, which have demonstrated that coffee is mainly a colouring agent, and not a potent erosive agent, owing to the deposition of tannins and pigments on enamel surfaces [11]. The high level of staining intensity in this group reflects pigment rather than mineral damage and is indicative of cosmetic rather than structural changes. The observations regarding mouthwash solutions apply only to the specific formulations tested and should not be generalised to all commercial products. Although the chlorhexidine mouthwash was responsible for mild erosion (3/10) and light staining, the neutral chlorhexidine-free mouthwash produced minimal changes (2/10) with no visible erosion. This agrees with previous research that has reported low inherent erosive activity of mouthwashes with a pH close to neutral [6]. The gentle staining associated with chlorhexidine is in accordance with other findings that reported an increase in the rate of chromogen fixation of chlorhexidine when combined with staining drinks [11].

On these matters, the results are qualitatively consistent with prior findings on beverage acidity and the changes in enamel surface; however, the qualitative nature of the assessment method, the author-developed scale, and the lack of intraoral simulation mean the results should be cautiously considered. Since quantification or mechanistic comparison is not employed, the results do not provide new evidence; instead, they offer a rather descriptive comparison of morphological changes within a controlled immersion model. Moreover, the in vitro design, the absence of salivary buffering, mechanical forces, and the limited scope of the studied beverages further limit direct clinical extrapolation. Thus, the scientific contribution must be taken on preliminary and exploratory terms.

The use of gentle agitation may enhance mass transport and reduce diffusion boundary layers, potentially accelerating enamel dissolution compared with static conditions. However, agitation was applied consistently across all groups to standardize exposure conditions and partially simulate oral fluid dynamics. Since the current model omits biofilm formation, carbohydrate metabolism, salivary pH cycling, and remineralisation, there is no implication for caries risk or clinical consequences. The current study does not seek to add methodological novelty or new mechanistic insight into enamel erosion. Instead, its contribution is to provide a structured qualitative comparison of beverage-induced enamel surface changes using a uniform, controlled static-immersion model.

### 4.1. Study Limitations

This study has a number of limitations that must be taken into consideration when evaluating its findings. The major drawback of the current study was its small sample size, with six individuals in each group. This small sample size limits the findings and makes it difficult to generalise them to the population at large. More substantial research is necessary to confirm the results. Initial surface roughness was not quantitatively standardized or measured prior to exposure, which may introduce minor baseline variability among specimens and represents a limitation of the study. The utilisation of a saliva substitute to mimic human saliva provides a reliable and consistent test environment, but it does not accurately simulate all the mechanisms of natural saliva, which include a protective action, the formation of a film on the teeth and the re-mineralisation of tooth enamel. Consequently, the actual clinical rate of erosion may be higher than the rate indicated by the experiment, implying that the results obtained should be considered in relative terms only rather than as a prediction of actual loss of tissue. The in vitro pH cycling model, involving five days of pH fluctuations, is an acute exposure scenario and does not replicate the in vivo processes, which are cumulative, adaptive and long-term. Tissue damage was assessed using semi-quantitative and qualitative techniques, including SEM and image analysis. The study did not evaluate titratable acidity, buffering capacity, or calcium/phosphate content of the tested beverages, which may influence erosive potential independently of pH and should be incorporated in future investigations. The in vitro data lack the complexity of real oral conditions, which involve salivary flow rates, the microbial population present in the mouth, mechanical wear, the impact of fluoride, along with varying behavioural and biological differences from one individual to another. Furthermore, the test specimens were exposed to a single drink, rather than the mixed diet that people typically consume. These results should be viewed as providing information on the comparative erosive and staining effects of dental products in a controlled environment, rather than as data that can be used to assess the actual risk that a patient will experience erosion or staining from a product.

From a translational perspective, the present erosion model may serve as a useful platform for evaluating preventive surface strategies aimed at enhancing enamel resistance to acidic challenges. Fluoride-based coatings, bioactive glass formulations, and calcium–phosphate releasing materials have demonstrated the capacity to promote remineralization and reduce mineral dissolution under erosive conditions. Future investigations could apply these protective treatments prior to acid exposure cycles to assess their ability to preserve enamel microstructure and limit prism dissolution observed in SEM analysis. Such approaches would allow direct comparison between untreated and protected enamel surfaces under standardized experimental conditions, contributing to the development of clinically relevant preventive protocols against dietary erosion.

### 4.2. Future Directions

Future research should more closely replicate the dynamic conditions of the oral environment. Models incorporating salivary flow, pellicle formation, pH cycling, and intermittent exposure to beverages would provide substantially greater physiological relevance than cyclic erosive exposure protocols. The inclusion of artificial saliva, rest periods, and controlled abrasion would further improve the simulation of clinical conditions. Expanding the range of tested beverages—including sugar-free products, sports and energy drinks, flavoured waters, herbal infusions, and plant-based alternatives—would offer a broader understanding of erosive potential across commonly consumed formulations. Acidic challenges were conducted at room temperature (23 ± 2 °C) to approximate typical beverage consumption conditions. Remineralization periods were performed at 37 °C in artificial saliva to simulate physiological intraoral temperature and salivary activity.

Additionally, evaluating the protective effects of remineralising agents such as fluoride, CPP–ACP, bioactive glass, or nanohydroxyapatite under standardised acidic challenges may contribute valuable insight into preventive strategies [31]. Finally, in situ and in vivo studies, despite their greater complexity, are essential for validating in vitro observations and for generating clinically meaningful, evidence-based guidelines to minimise enamel erosion in real-life scenarios. Future studies should incorporate validated quantitative assessments, cyclic and clinically realistic exposure protocols, standardised imaging, reproducible scoring systems, and controlled statistical designs to provide mechanistic and clinically applicable evidence.

## 5. Conclusions

Within the limitations of this in vitro erosive pH-cycling model, acidic beverages were associated with progressively greater enamel surface alterations, as demonstrated by both qualitative SEM observations and quantitative grayscale texture analysis. Citric-acid–containing solutions produced the most pronounced microstructural changes, followed by phosphoric-acid beverages, whereas coffee and mouthwash solutions induced minimal surface modification.

The observed differences should not be attributed solely to beverage pH. Enamel erosion is a multifactorial process influenced by additional physicochemical properties, including titratable acidity, buffering capacity, chelating effects of organic acids, and mineral ion composition, which were not evaluated in the present study. Therefore, the findings should be interpreted as comparative morphological responses under controlled experimental conditions rather than direct predictors of clinical erosion severity.

The integration of SEM imaging with ImageJ-based quantitative texture analysis provides an accessible and reproducible approach for objective assessment of enamel surface changes in laboratory models. Future studies combining morphological, mechanical, and compositional analyses are needed to better clarify the mechanisms underlying beverage-induced enamel erosion and to improve clinical translation.

## Figures and Tables

**Table 1:** Test solutions used in the in vitro immersion protocol.

Code	Test Solution	Type	Measured pH (Mean ± SD)
RCC	Coca-Cola (Coca-Cola HBC, Timisoara, Romania)	Phosphoric acid-based carbonated soft drink	2.52 ± 0.03
RL	Lemon juice (Merlin’s Lemonade No. 1 Lemon, Merlins Beverages, Bucharest, Romania)	Citric acid-based lemonade	3.70 ± 0.04
RP	Orange juice (Tropicana Orange Juice No Pulp, Tropicana Brands Group, Chicago, IL, USA)	Citric acid-containing orange fruit juice (no pulp)	4.78 ± 0.05
RC	Coffee (Costa Coffee Signature Blend Medium Roast, Costa Coffee, London, UK)	Brewed coffee beverage	5.18 ± 0.06
RAC	Mouthwash with 0.12% chlorhexidine (Perio-Aid Intensive Care 0.12%, Dentaid, Barcelona, Spain)	Antimicrobial mouthwash	5.52 ± 0.04
RA	Mouthwash without chlorhexidine (Curaprox Perio Plus Zero, Curaden AG, Kriens, Switzerland)	Non-antimicrobial fluoride mouthwash	6.81 ± 0.05

**Table 2:** SEM tooth wear assessed in light of the Basic Erosive Wear Examination index created by Bartlett.

Grade 0 (No Erosion; Corresponding to BEWE 0)	Grade 1—Minimal Erosion	Grade 2 (Mild Erosion; Corresponding to BEWE 1–2)	Grade 3 (Moderate Erosion; Corresponding to BEWE 2)	Grade 4 (Severe Erosion; Corresponding to BEWE 3)	Grade 5 (Extreme Erosion; Corresponding to Advanced BEWE 3 with Dentine Expo-Sure)
- No erosion at all of the enamel. - Enamel surfaces are smooth and healthy, with no signs of decay. - The SEM view reveals that the surface possessed a glossy appearance, featuring enamel crystals that were either very clearly visible or hard to make out. The material between the crystals remained unchanged.	- Very slight erosion, mainly that which is expected from everyday normal weathering processes. - The surface is fairly flat; however, it does not have its usual shine at lower power. - Minimal surface modifications resulted in the prismatic structure remaining intact, while the prism’s borders became apparent.	- Minor damage. - Localised alterations. - Small areas that showed the start of a texture reminiscent of honeycomb.	- Noticeable erosion effect. - The surface exhibits pronounced development of enamel rods, along with considerable loss of interrod enamel. - A lot of the crystals in this location showed that they had been broken down into hexagonal sections, evidence that they had been subjected to erosion. - Hexagonal depressions indicated dissolution of prism core material.	- Severe erosion. - The prismatic structure is heavily damaged. - The widespread destruction of the original prism architecture resulted in a honeycomb pattern caused by the substantial loss of interprismatic substance. The prism cores dissolved completely with considerable surface cracks.	- Enamel is heavily eroded, classed as severe. - The enamel here was almost entirely worn away. Extensive damage to the prismatic architecture is noted in the form of considerable deep cratering on its surface. This tooth had a very uneven and gritty texture; it was also noticeably discoloured, showing where bone had been lost, and with dentine visible.

**Table 3:** Quantitative grayscale texture analysis of enamel surfaces following immersion in tested solutions (ImageJ analysis).

Group	Tested Solution	Mean Gray Value (Mean ± SD)	StdDev Gray Value	Surface Interpretation
Control	Artificial saliva (control)	83.70 ± 3.1	33.18	Natural enamel microtopography
RC	Coffee	87.52 ± 2.2	36.41	Mild surface alteration, mainly staining
RCC	Coca-Cola	94.31 ± 1.9	42.87	Pronounced erosive surface changes
RP	Orange juice	91.35 ± 2.7	39.58	Moderate erosive surface changes with partial prism exposure
RL	Lemon juice	98.12 ± 2.4	45.76	Severe enamel demineralization
RA	Mouthwash without chlorhexidine	79.20 ± 2.2	31.86	Minimal surface modification
RAC	Mouthwash with CHX	75.27 ± 1.8	29.47	Minimal surface modification

**Table 4:** Descriptive distribution of beverage groups, enamel alteration categories, and staining intensity (n = 36).

Beverage	Code	pH	Enamel Erosion Description	Staining/Colour Change
Coca-Cola	RCC	2.52	“Severe–extreme erosion; pronounced honeycomb pattern; deep craters; highest grade”	“Yellow–brown”
Lemon juice	RL	3.70	“Most severe destruction; near-complete loss of prism structure; deep irregular patterns”	“Whitish”
Orange juice	RP	4.78	“Moderate–severe erosion; clear prism exposure; pits; rough”	“Chalky surface”
Coffee	RC	5.18	“Minimal structural change; prisms largely preserved; slight surface alteration”	“Uniform brown staining”
Mouthwash with chlorhexidine	RAC	5.52	“Mild erosion; slight surface change; low-grade damage”	“Thin whitish cervical residues; slight staining”
Mouthwash without chlorhexidine	RA	6.81	“Minimal surface alterations without features characteristic of erosive enamel loss”	“Almost unchanged; very slight whitening”

## Data Availability

All data regarding this manuscript can be requested from the corresponding authors: marian.diana@uvvg.ro and cojocariu.carolina@uvvg.ro.
